# Massachusetts

**Published:** 1896-06

**Authors:** 


*Massachusetts.* Reports come to us from the Food Commissioners showing a general improvement in the whole milk-supply of the State, less disposition to adulterate, color, or add injurious preserving agents, because milk taken from healthy herds in good sanitary condition and clean surroundings will keep in good condition a sufficient period of time to permit its sale at a profitable price. Let the agitation for a pure and whole some food-supply go on, and many more such reports will annually come to us in the future.

In Boston a rabid dog produced a panic among the children in a school-yard, and succeeded in biting one of them before being killed. An adult was also bitten before the dog reached the school.

